# Modelling local patterns of child mortality risk: a Bayesian Spatio-temporal analysis

**DOI:** 10.1186/s12889-020-10016-9

**Published:** 2021-01-06

**Authors:** Alejandro Lome-Hurtado, Jacques Lartigue-Mendoza, Juan C. Trujillo

**Affiliations:** 1 Economics Department, Universidad Autónoma Metropolitana, Unidad Azcapotzalco, Av. San Pablo 180, Col. Reynosa Tamaulipas, Alcaldía Azcapotzalco, C.P, 02200 CDMX, Mexico; 2 Universidad Anáhuac México, Economics and Business School, Avenida de las Torres 131, Colonia Olivar de los Padres, C.P, 01780 Ciudad de México, Mexico; 3 Department of Environment and Geography, University of York, York, North Yorkshire YO10 5NG UK

**Keywords:** Children’s health, Bayesian mapping, Child mortality risk, Space-time interactions, Mexico

## Abstract

**Background:**

Globally, child mortality rate has remained high over the years, but the figure can be reduced through proper implementation of spatially-targeted public health policies. Due to its alarming rate in comparison to North American standards, child mortality is particularly a health concern in Mexico. Despite this fact, there remains a dearth of studies that address its spatio-temporal identification in the country. The aims of this study are i) to model the evolution of child mortality risk at the municipality level in Greater Mexico City, (ii) to identify municipalities with high, medium, and low risk over time, and (iii) using municipality trends, to ascertain potential high-risk municipalities.

**Methods:**

In order to control for the space-time patterns of data, the study performs a Bayesian spatio-temporal analysis. This methodology permits the modelling of the geographical variation of child mortality risk across municipalities, within the studied time span.

**Results:**

The analysis shows that most of the high-risk municipalities were in the east, along with a few in the north and west areas of Greater Mexico City. In some of them, it is possible to distinguish an increasing trend in child mortality risk. The outcomes highlight municipalities currently presenting a medium risk but liable to become high risk, given their trend, after the studied period. Finally, the likelihood of child mortality risk illustrates an overall decreasing tendency throughout the 7-year studied period.

**Conclusions:**

The identification of high-risk municipalities and risk trends may provide a useful input for policymakers seeking to reduce the incidence of child mortality. The results provide evidence that supports the use of geographical targeting in policy interventions.

## Background

There is a public concern regarding the high percentage of child mortality. Globally, there were 5.6 million child deaths during 2016 [1]. As a consequence, the world has witnessed an increasing policy interest in improving children’s health, reflected in the United Nations’ third Sustainable Development Goal (SDG) on good health and wellbeing; particularly, in its aim to end preventable deaths of new-born and children under five by the year 2030 [2].

Between 2010 and 2017, under-five mortality rate (per 1000 live births) decreased from 17.4 to 13.4 in Mexico [3]. However, these numbers are still higher than those observed in North American developed countries. For instance, during the referenced years these rates declined from 7.3 to 6.6 in the United States and from 5.6 to 5.1 in Canada [3]. Similarly, the probabilities of dying at age 5–14 years (per 1000 children age 5) in Mexico were 2.8 in 2010 and 2.5 in 2018, while in the United States these figures were 1.3 for both years, and in Canada 1.1 and 1.0, respectively [4]. Similarly, data from the World Bank indicate that Mexican infant mortality rate (per 1000 live births) reduced from 14.9 in 2010 to 11.6 in 2017 [5]. Nevertheless, such rates are still high in comparison to the United States and Canada, where the figures declined, during the aforesaid years, from 6.2 to 5.7, and from 4.9 to 4.4, respectively.

This paper focuses on the modelling of child mortality risk trends across different geographical areas in Greater Mexico City for the first time, allowing contribution to the existing literature [6–9] on the spatial analysis of such risk. Gayawan et al. [6] illustrated the regional variations of child mortality among ten West African countries, finding some clusters of higher child mortality in northwest and northeast Nigeria. Jimenez-Soto et al. [7] showed the disparities among child mortality across rural-urban locations and regions in Cambodia, and analogous findings, additionally including variations within inter and intra regions, were made in Papua, New Guinea [8]. In Mexico, a similar study [10] analyzed the child mortality trend caused by diarrhea in all Mexican states, identifying different spatial patterns of the peak mortality rate across time.

The relevance of considering the potential spatial structure of the data is grounded on the fact that communities are often clustered with respect to certain shared characteristics, such as their socioeconomic background [11]. Presumably, people with a high socioeconomic status live close to each other, and likewise among other socioeconomic standings [12]. However, socioeconomic status is not the sole factor underlying child mortality; if the availability of data allows it, other variables, such as environment, urbanization, or the genetics of people, must be regarded in a spatial analysis [11, 13, 14]. McDonald et al. [13], analyzing American counties located in the US-Mexican border, found urbanization level as the most relevant variable for explaining child mortality, while ethnicity –Hispanic or non-Hispanic white– appeared to be less relevant. According to these last authors, higher mortality rates in non-metropolitan communities were attributed to a diminished access to emergency and special care facilities, limited emergency medical service capabilities, as well as fewer health care providers per capita. Castro-Ríos et al. [15] found that access to social security increases the surviving probability of children with accute lymphoblastic leukemia. More accurately, the research concluded that children who had been insured for less than half of their lives had more than a twofold risk of death than children insured throughout their entire lives.

Child mortality may not only vary over space but also over time, as it has been determined in previous health studies; such is the case of the spatio-temporal variations of stomach cancer risk [16] and asthma risk [17]. Besides, people’s health risks may vary over space and time due to changes in health-related behaviours, namely physical activity, smoking, and diet [18]. Thus, in order to gain a better understanding, the need for analyzing not just the spatial pattern of the mortality risk but also its local trend over time, at the geographical level, becomes evident. Therefore, this study uses a Bayesian modelling approach [19] owing to the space, time, and space-time structure of the data, while the methodology is based on random effects, which enables the modelling of the geographical variation of children mortality over time. It must be acknowledged that this methodology has been used in the area of criminology [20].

The aims of this study are (i) to model the evolution of child mortality risk at the municipality level in Greater Mexico City, (ii) to identify municipalities with high, medium, and low risk over time, and (iii) using local trends, to ascertain potential high-risk municipalities.

## Methods

### Area of study and child mortality data

Greater Mexico City, one of the most populated urban areas in the world, is the third-largest metropolis among the Organisation for Economic Co-operation and Development (OECD) countries and the world’s largest outside of Asia [21]. It consists of 16 municipalities within Mexico City and 59 in the State of Mexico^1^ (see figure 4 in the Appendix). According to the Mexican National Institute of Statistics and Geography (INEGI) [22], it had 20,892,724 inhabitants, covering a land area of 7866 km^2^ in 2015. In economic terms, it is considered the most important metropolitan area in Mexico, accounting for 25% of the country’s gross domestic product in 2017 [23]. The present study explored the catalogs of death and birth records, issued by the Mexican Ministry of Health, of 75 municipalities within Greater Mexico City in a period spanning from January 2011 to December 2017. This study considered children from 0 to 5 years old [24]. During the study period, the total number of live births in the region was 2,121,601, while the number of deaths of children totalled 35,862. The raw data (death and birth records) from Mexican Ministry of Health [25] was aggregated in order to perform a spatial and temporal analysis at the municipality level.

### Statistical analysis

**Fig. 1:**
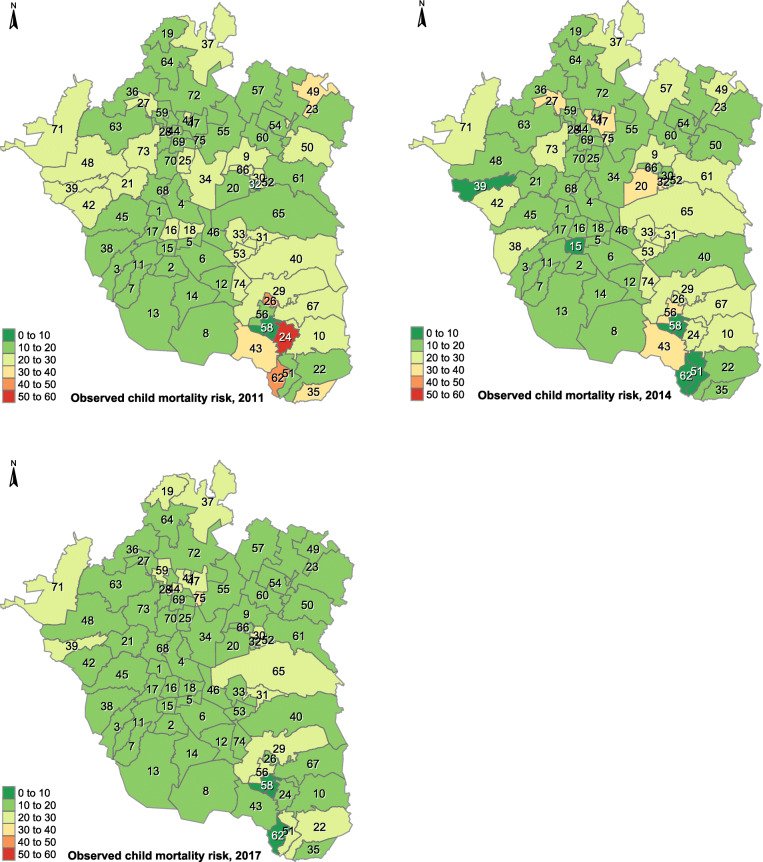
Geographical pattern evolution of the observed child mortality risk (per 1000 live births) in Greater Mexico City. Figure 1 depicts the temporalevolution of the geographical pattern of the observed child mortality risk (per 1000 live births) in Greater Mexico City, at the start (2011), middlef1:3 (2014), and end (2017) of the study period. Source: Own elaboration using data from INEGI and Mexican Ministry of Health

xPreliminary analyses were carried out to investigate the potential presence of spatial autocorrelation and serial correlation of the data using the Global Moran Index [26] and the Autocorrelation Function (ACF). Furthermore, a Bayesian spatio-temporal model was used to model the potential spatial and temporal child mortality risk. This research assumes the number of child deaths, *y*_*it*_, in the municipality *i*, at a given period *t* (=2011, 2013, …, 2017) follows a binomial distribution [27], *y*_*it*_~*Binomial*(*n*_*it*_, *μ*_*it*_), where *n*_*it*_ represents the total number of live births in the municipality *i* at period *t*; and *μ*_*it*_ denotes the child mortality risk in the municipality *i* at period *t*. According to Law et al. [28] and Li et al. [20], child mortality risk can be modelled as:

1 $$ logit\left({\mu}_{it}\right)=\alpha +\left({s}_i+{u}_i\right)+{d}_0{t}^{\ast }+{v}_t+{d}_{1i}{t}^{\ast }+{\varepsilon}_{it} $$

where α is the overall logit child mortality risk across the 7-year period and the terms *s*_*i*_ and *u*_*i*_ are the spatial components, which capture the spatial structure and unstructured effects of the data, respectively. These components are common among the study period and account for the level of clustering and the heterogeneity of the data, respectively. We assigned a Gaussian distribution to the spatially unstructured random effect term, *u*_*i*_. Additionally, *d*_0_*t*^∗^ + *v*_*t*_ is the overall time trend in all of the municipalities. The first term (*d*_0_*t*^∗^) assesses the linear trend, and the second (*v*_*t*_), with additional Gaussian noise, allows for nonlinearity in the overall trend pattern; *v*_*t*_ follows a normal distribution, ${v}_t\sim N\left(0,{\sigma}_v^2\right)$. Note that *t*^∗^ is centered at the mid observation period, *t*^∗^ = *t* − 4. The expression *d*_1*i*_*t*^∗^ denotes the spatio-temporal structure of the data, which permits each municipality to have a different trend from the overall time trend-pattern. This term plays an important role considering that child mortality trends exhibit variability at the local level (see Fig. 1). Thus, *d*_1*i*_*t*^∗^ represents, and assumes, a linear departure of the municipality temporal trend from the common trend; such local trend can have an increasing, decreasing, or stable tendency from the overall linear pattern. Finally, ${\varepsilon}_{it}\sim N\left(0,{\sigma}_{\mathcal{E}}^2\right)$ is the component addressed to contain the variability that is not explained by other terms, and may include overdisperfsion; that is, when the variation of the data is higher than its mean –a common characteristic of binomial models [29, 30].

We assigned the BYM (Besag, York, and Mollié) model to the structured spatial components (*s*_*i*_ *and d*_*i*_) [31]. Following previous studies [19, 20], we allocated an intrinsic conditional autoregressive Gaussian distribution (ICAR) to the priors of the spatial structure (*s*_*i*_) and the spatio-temporal interaction term (*d*_*i*_). Thus, the terms *d*_*i*_ and *s*_*i*_ depend on the neighbouring areas, meaning that near areas are more likely to have similar values; in our specific case, it means that nearby municipalities are assumed to have similar child mortality risk rates. This is controlled by a spatial adjacency matrix *W* of size *N x N,* where the diagonal values are *wii = 0* and the off-diagonal entries are *wij = 1* if municipalities *i* and *j* share a common boundary, otherwise *wij = 0*. In this sense, if two municipalities are defined to be neighbours, their random effects are correlated, otherwise they are conditionally independent. The conditional expectation of *d*_*i*_ is equal to the mean of the random effects in neighbouring municipalities, whereas the conditional variance is inversely proportional to the number of neighbour municipalities; this is similar for *s*_*i*_. Note that *d*_*i*_ may also control for the potential endogeneity due to the interaction between space and time. In accordance with previous studies’ proceedings [20], we allocated a hyperprior distribution of Gamma, a highly non-informative distribution [32], on the variance of *d*_*i*_, *s*_*i*_, *u*_*i*_, *v*_*t*_, and *ε*_*it*_. Finally, following the Gelman criterion [33], all random effect standard deviations, such as *σ*_*d*_, *σ*_*s*_, *σ*_*u*_, *σ*_*v*_, and *σ*_*ϵ*_, have a positive half Gaussian prior *N*_+∞_(0, 10).

To classify each municipality as a high, low, or medium-risk municipality across time, we used the values of the posterior probability of the spatial component *p*(exp(*u*_*i*_ + *s*_*i*_) > 1|*data*). Posterior probability refers to the probability of getting a specific value of the spatial component; in this case, that the value is greater than 1 (meaning that such municipality would have an excess of mortality). The component *exp*(*u*_*i*_ + *s*_*i*_) indicates the average odds for each municipality, during the study period, with respect to the overall mortality odds. Thus, the values greater than 0.8, between 0.2 and 0.8, and lower than 0.2 were classified as high, medium, and low-risk municipalities, respectively. This first classification can be expressed in the *hi* term which is equal to *1* for a high-risk area, *2* for a low-risk area, and *3* for a medium-risk area. This criterion has been used in previous studies [31]. Further, to measure the local trend of each classified municipality, for each specific category *hi* we used the values of the posterior probability of the local slopes, *d*_1*i*_. This technique enabled the measurement of each municipality’s local dynamic pattern throughout the study period. Thus, if *p*(*d*_1*i*_ > 0|*hi*, *data*) > 0.8, *p*(*d*_1*i*_ > 0|*hi*, *data*) < 0.2, or 0.2 < *p*(*d*_1*i*_ > 0|*hi*, *data*) < 0.8, the municipality was classified as having an increasing, decreasing, or stable trend, in comparison with the overall trend.

The model was implemented in R [34] and WinBUGS [35] (statistical software). We ran MCMC chains of 100,000 (for this number, the model reaches convergence) with different initial values. 70,000 iterations were used for making inferences from the model, after having burned in the first 30,000. The convergence was examined by visual inspection of the history plots and through the Gelman-Rubin diagnostic [33], which are standard statistical tests to measure convergence of MCMC chains. The values from the Gelman-Rubin diagnostic remained lower than 1.04 for every single model parameter, meaning that the chains achieved convergence after the burn-in period.

## Results

### Descriptive analysis

The Global Moran Index of the data for each year was positive and significant, with a mean value of 0.32 and a *p* value < 0.0001, illustrating the presence of a positive spatial correlation in the records. In other words, this result is indicative of the existence of some nearby municipalities with a similar mortality risk. The ACF mean was 0.58 (lagged 1 year for each municipality) across all the municipalities. This number evidences the presence of serial correlation; that is, the association of certain level of observed mortality across time.

Table 1 provides descriptive statistics of the observed child mortality rate in Greater Mexico City, from 2011 to 2017, defined as the number of deaths of children, aged 0 to 5 years, per 1000 live births. Broadly, there was a slight mitigation on the average child mortality rate during these years, shifting from 20.4 in 2011 to 17.47 in 2017. Note that according to the means and variances, illustrated in Table 1, a trace of overdispersion was present in our data.

**Table 1:** Descriptive statistics. Observed child mortality rate^a^ in Greater Mexico City (2011 to 2017)

Observed child mortality risk	2011	2012	2013	2014	2015	2016	2017
Mean	20.4	19.91	18.42	18.12	18.97	17.08	17.47
Standard deviation	8.18	7.03	6.09	6.39	8.87	7.41	5.26
Minimum value^b^	1.92	1.49	2.16	2.32	2.22	1.74	3.3
Maximum value^c^	58.39	51.02	35.64	34.3	60.34	55.56	35.71

^a^Child mortality rate = (child deaths / number of resident live births) × 1000. In other words, number of child deaths per 1000 resident live births

^b^Corresponds to the municipality with the minimum value among all the municipalities of Greater Mexico City

^c^Corresponds to the municipality with the maximum value among all the municipalities of Greater Mexico City

Figure 1 depicts the temporal evolution of the observed child mortality risk by municipality in Greater Mexico City at different years − 2011, 2014, and 2017 (start, middle, and end)– throughout the study period. The dark green and red colors indicate the lowest and highest risk, respectively. Overall, municipalities with higher risk were enclosed in the east, besides a few scattered in the north. Conversely, municipalities with the lowest mortality risk clearly constituted a cluster in the south-west (darker green color).

### Modelling spatial-temporal patterns

**Fig. 2:**
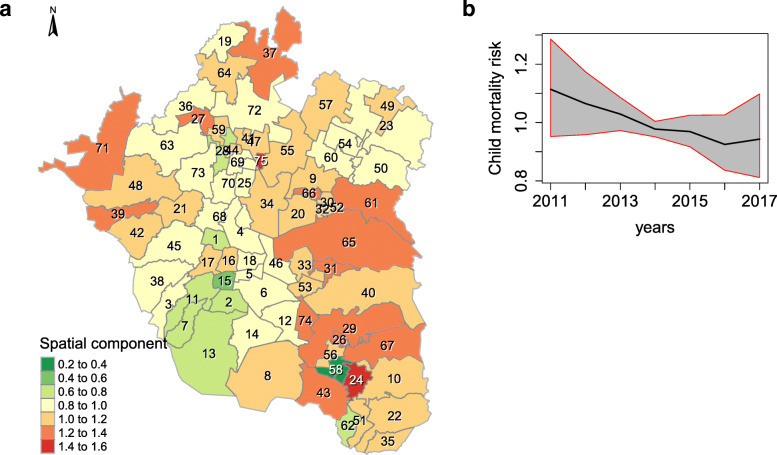
**a** and **b**. Spatial child mortality risk, at the municipality level, and its overall trend in Greater Mexico City. **a** shows the spatial component of the child mortality risk during the study period. Those areas with risk values greater or lower than 1 have a higher or lower child mortality risk in comparison with the average, respectively. **b** displays the overall risk trend, with a 95% CI, from 2011 to 2017. Source: Own elaboration using data from INEGI and Mexican Ministry of Health

Figure 2a illustrates the child mortality risk by municipality, compared to the average throughout the study period. A risk value above or below 1 suggests a higher or lower risk associated with the concerning municipality, in comparison to the Greater Mexico City 7–year average. The figures mainly display that municipalities menaced by a higher child mortality risk are those situated in the surroundings of Mexico City: the east area, along with a few in the north. Concurrently, municipalities located in Mexico City, as well as a few in the north of the studied area, presented lower risks. Figure 2b illustrates the overall time trend of the relative risk in comparison to the Greater Mexico City average from 2011 to 2017. It is possible to observe, overall, a slightly decreasing tendency of such risk.

**Fig. 3:**
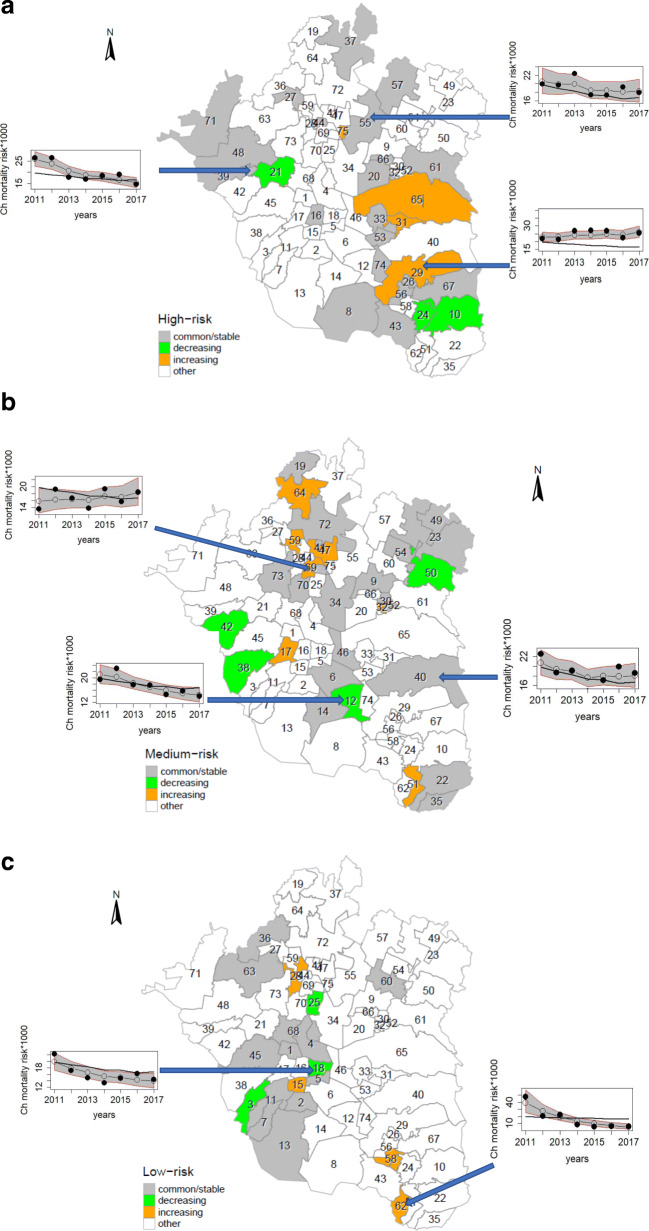
**a**. Temporal trend in child mortality risk for high-risk municipalities. **a** displays the temporal dynamics of child mortality risk for high-risk municipalities in Greater Mexico City, which are classified into 3 categories: stable, decreasing, and increasing risk. The inserted figures show the observed child mortality risk (black solid dots), the estimated child mortality risk –posterior mean of risk– (open circles and dashed line) with a 95% CI (grey region), and the estimated common trend (black line) over time. Source: Own elaboration using data from INEGI and Mexican Ministry of Health. **b**. Temporal trend in child mortality risk for medium-risk municipalities. **b** displays the temporal dynamics of child mortality risk for medium-risk municipalities in Greater Mexico City, which are classified into 3 categories: stable, decreasing, and increasing risk. The inserted figures show the observed child mortality risk (black solid dots), the estimated child mortality risk –posterior mean of risk– (open circles and dashed line) with a 95% CI (grey region), and the estimated common trend (black line) over time. Source: Own elaboration using data from INEGI and Mexican Ministry of Health. **c**. Temporal trend in child mortality risk for low-risk municipalities. **c** displays the temporal dynamics of child mortality risk for low-risk municipalities in Greater Mexico City, which are classified into 3 categories: stable, decreasing, and increasing risk. The inserted figures show the observed child mortality risk (black solid dots), the estimated child mortality risk –posterior mean of risk– (open circles and dashed line) with a 95% CI (grey region), and the estimated common trend (black line) over time. Source: Own elaboration using data from INEGI and Mexican Ministry of Health

Figure 3a, b, and c display the temporal dynamics of high-risk, medium-risk, and low-risk municipalities. Of the total, high-risk municipalities amounted to 27 (36%), medium-risk to 29 (39%), and low-risk to 19 (25%). On the whole, high-risk municipalities were located in the east of the metropolitan area along with a few spots in the north and west (see Fig. 3a). Meanwhile, most of the low-risk municipalities were located in the south-west (Fig. 3c). Finally, medium-risk municipalities were mostly situated in the north, in addition to a few of them scattered in the east, center, and west, as exhibited in Fig. 3b. All of these classified municipalities were significant at the 95% Credible Interval (CI).

The inserted small graphs in Fig. 3a, b, and c show the different trends of the observed risk (black solid dots), the estimated risk (dashed line with open circles) with 95% CI (grey region), and the estimated common trend (black line) of the mortality risk over time.

Figure 3a exhibits that most of the high-risk municipalities (74%) had a stable dynamic regarding their child mortality risk trend; whereas, 4 of them (15%) showed an increasing trend. The remaining 3 high-risk municipalities (11%) were the only ones with a decreasing trend over time. Figure 3b depicts medium-risk municipalities, 24% of which exhibited an increasing trend in risk. The majority of the medium-risk municipalities (62%) presented a stable tendency, whereas municipalities with a decreasing trend amounted to 14%.

Finally, Fig. 3c illustrates that 21% of low-risk municipalities experienced a relative increment in child mortality risk over time. However, most of these low-risk municipalities (63%) had a stable trend during the study period, leaving just 16% of them under the category of decreasing trend.

## Discussion

This research studied child mortality dynamics across municipalities in Greater Mexico City. It identified municipalities with high child mortality risk, as well as those with medium risk that, given their trend, may become high risk.

**Fig. 4:**
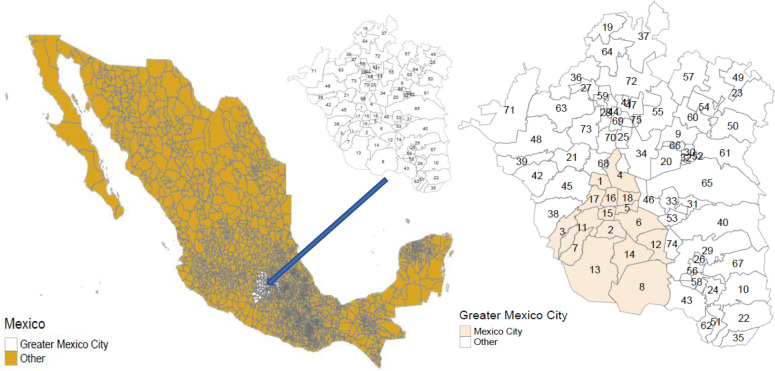
Mexico, Greater Mexico City, and Mexico City. Municipalities of Greater Mexico City: 1) Azcapotzalco, 2) Coyoacán, 3) Cuajimalpa de Morelos, 4) Gustavo A. Madero, 5) Iztacalco, 6) Iztapalapa, 7) La Magdalena Contreras, 8) Milpa Alta, 9) Acolmán, 10) Amecameca, 11) Alvaro Obregón, 12) Tláhuac, 13) Tlalpan, 14) Xochimilco, 15) Benito Juárez, 16) Cuauhtémoc, 17) Miguel Hidalgo, 18) Venustiano Carranza, 19) Apaxco, 20) Atenco, 21) Atizapán de Zaragoza, 22) Atlautla, 23) Axapusco, 24) Ayapango, 25) Coacalco de Berriozábal, 26) Cocotitlán, 27) Coyotepec, 28) Cuautitlán, 29) Chalco, 30) Chiautla, 31) Chicoloapan, 32) Chiconcuac,33) Chimalhuacán,34) Ecatepec de Morelos, 35) Ecatzingo, 36) Huehuetoca, 37) Hueypoxtla, 38) Huixquilucan, 39) Isidro Fabela, 40) Ixtapaluca, 41) Jaltenco, 42) Jilotzingo, 43) Juchitepec, 44) Melchor Ocampo, 45) Naucalpan de Juárez, 46) Nezahualcóyotl, 47) Nextlalpan, 48) Nicolas Romero, 49) Nopaltepec, 50) Otumba, 51) Ozumba, 52) Papalotla, 53) La Paz, 54) San Martín de las Pirámides, 55) Tecámac, 56) Temamatla, 57) Temascalapa, 58) Tenango del valle, 59) Teoloyucan, 60) Teotihuacan, 61) Tepetlaoxtoc, 62) Tepetlixpa, 63) Tepotzotlán, 64) Tequixquiac, 65) Texcoco, 66) Tezoyuca, 67) Tlalmanalco, 68) Tlalnepantla de Baz, 69) Tultepec, 70) Tultitlan, 71) Villa del Carbón, 72) Zumpango, 73) Cuautitlán Izcalli, 74) Valle de Chalco Solidaridad and 75) Tonanitla. Source: Own elaboration using data from INEGI

**Fig. 5:**
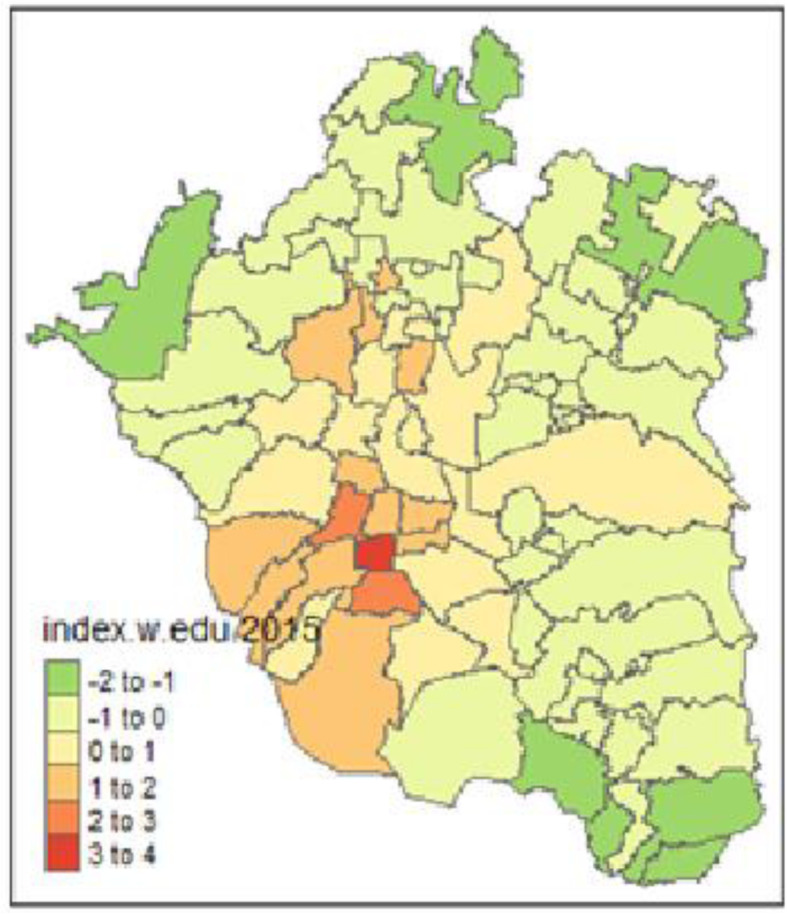
Spatial distribution by economic and education level as of 2015. Figure 5 displays the economic and education index across Greater Mexico City as of 2015. The dark green and red colors depict the municipalities with the worst and best conditions in these issues, respectively. Source: Own elaboration using data from INEGI, 2015 [21]

Our findings illustrate that 36% of the total municipalities fit into a high-risk categorization. These municipalities are, overall, located in the east of Greater Mexico City, along with a few spots in the north and west. This is an expected result given that Mexico City (see Fig. 4 in the Appendix) holds the best economic and socioeconomic conditions, while the surrounding municipalities have lower economic and social standings. According to INEGI, the north, west, and east areas of Greater Mexico City are characterized by their relatively lower socioeconomic and education levels with respect to the average (see Figure 5 in the Appendix). The previous results are in line with Sreeramareddy et al. [36] and Aheto [37], who identify a positive association between deprived economic conditions and child mortality risk; the lower the level of income, the greater the probabilities of higher child mortality rates.

In terms of risk evolution, four high-risk municipalities (Chalco, Chicoloapan, Texcoco, and Tonanitla), mostly located in the west, exhibited an increasing trend over time. Similarly, seven medium-risk municipalities (Miguel Hidalgo, Chiconcuac, Nextlalpan, Ozumba, Teoloyucan, Tequixquiac, and Tultepec), accounting for 24% of the total in that category, manifested an increasing trend over time, representing their liability to become high risk in the short term. Six of these seven municipalities are in the north area. As Figure 5 in the Appendix depicts, these municipalities with high and medium risk face deprived economic and social conditions, with the exception of Miguel Hidalgo. These results are congruent with those of Escamilla-Santiago et al. [38], who evidenced, for the period between 1990 and 2009, an increasing cancer mortality rate in children and teenagers residing in high marginalized Mexican states.

It must be acknowledged that a decreasing trend manifested in the average mortality risk likelihood over the 7-years study period. This result coincides with Aguirre and Vela-Peóns work [39], who, by deploying the brass mortality method, estimated a decreasing infant mortality rate in Mexico from 1990 to 2010. This slight decrement may partially be explained as the result of diverse public health policies, such as the public programmes deployed by the Mexican Ministry of Health aimed at decreasing neonatal mortality risk. Among these programmes, it is worth mentioning “Programa de Acción: Arranque Parejo en la Vida, 2002”, “Programa de Acción Específico 2007-2012, 2008”, and “Programa de Acción Específico Salud Maternal y Perinatal, 2013-2018” [40].

Finally, it should be acknowledged that owing to data limitations, the results here exposed require a word of caution. Specifically, we assumed no mobility of children. Although this assumption may not apply in a dynamic area such as Greater Mexico City, more precise data –unavailable at the time– would be required to permit the consideration of this factor. Consequently, as in other studies [41, 42], the mobility of people was not included. Despite the previous constraint, we hope the key strengths of this study, including space, time, and space-time structures, may provide relevant insights for diminishing child mortality risk in Greater Mexico City. In this sense, McLaughlin et al. [43] highlighted the importance of spatial data and the local context, as inputs for policy decisions. Likewise, in the area of health, Ugarte et al. [44] illustrated how spatial and temporal trends provide useful information for addressing health inequalities. However, in order to complement this study, future studies should aim to investigate additional potential factors underlying the mortality of children.

## Conclusion

By unearthing the identification and evolution of child mortality risk on municipalities belonging to Greater Mexico City, the findings of this research may provide an important input for policy decisions addressed to reduce the mortality of children. Locations with high child mortality risk should be benefited from priority interventions. In this sense, this analysis provides important baseline information for decision-makers. The identification of spatial and temporal trends across different areas supplies decision-makers with relevant inputs for designing programmes to tackle health inequalities [45]. Using these inputs, spatially-targeted programmes may focus on small locations, allowing policy measures to have a more effective local impact. In this regard, it has already been demonstrated that in comparison with programmes where resources are not addressed towards specific geographical areas [45, 46], vulnerable and local groups benefit more when the aforementioned inputs are used. This study identified municipalities with medium and high child mortality risk, especially those with an increasing trend over time (Chalco, Chicoloapan, Texcoco, and Tonanitla, in the case of high child mortality risk), which helps to implement the geographical targeting of policy efforts to reduce it. Given the overall scarcity of healthcare resources in Mexico, we hope these results may contribute to the improvement of cost-effective policies.

## Data Availability

Raw data are freely available at (open access): http://www.dgis.salud.gob.mx/contenidos/basesdedatos/da_defunciones_gobmx.html [25].

## Abbreviations

SDG: Sustainable Development Goal
US: United States of America
OECD: Organisation for Economic Co-operation and Development
ACF: Autocorrelation Function
BYM: Besag, York, and Mollié
ICAR: Intrinsic Conditional Autoregressive
MCMC: Markov Chain Monte Carlo
CI: Credible Interval
INEGI: National Institute of Statistics and Geography

## Footnotes

1 An additional municipality, excluded from this analysis, belongs to the State of Hidalgo.
